# Vitamin D3 and its necessity for health

**DOI:** 10.1016/j.amsu.2022.103872

**Published:** 2022-05-26

**Authors:** Safinaz Khan, A.H.M. Ataullah, Sabrina Rahman, Md Moshiur Rahman

**Affiliations:** Department of Biochemistry and Molecular Biology, Sir Salimullah Medical College, Dhaka, Bangladesh; Medical Officer, Sher-E-Bangla Medical College Hospital, Barishal, Bangladesh; Department of Public Health, Independent University-Bangladesh, Dhaka, Bangladesh; Neurosurgery Department, Holy Family Red Crescent Medical College, Dhaka, Bangladesh

Vitamin D, a fat soluble vitamin plays a crucial role in micronutrient (calcium and phosphate) homeostasis, also engaged in bone metabolism. Vitamin D can be found in different chemical forms. VitaminD3-cholecalciferol can be ground in fish, fish oil,liver, meat.In addition, skin can also form vitamin D3 in adequate sun exposure due to UV-B subjection. Storage form and active form is 25-OH D3 and 1,25-(OH)2D3 respectively. Vitamin D2(ergocalciferol) is ingested from plants(wild mushrooms).Recommended vitamin D allowance for a healthy subject is 600–800 IU in vitamin D3 formulation [1].

Isomers of Vitamin D content in breast milk is very negligible.As a result, through breastfeeding, infants get less than 20% of recommended dietary allowance of vitamin D.This has been proved that,96% of children having bone demineralization, e.g.- rickets had only breastfeeding.In 2008, The American Academy of Pediatrics advised a supplementation of 400IU of vitamin D along with exclusive or partly breastfed babies to some extent from the beginning of life.In intestinal malabsorption, vitaminD therapy showed better result in rickets and reduce serum alkaline phosphatase level [2]. Vitamin D receptors(VDR) are found in a large scale throughout human bodies, which results in some extraskeletal manifestations in decreased vitamin D condition.To meetup desired serum concentration of vitamin D3, fortification and supplementation is necessary [3,4].

Vitamin D boosts up innate and adaptive immune system by expressing VDR in different white blood cells, scavenger cells to mitigate tumor proliferation, invasion and metastasis by induction of programmed cell death, cell differentiation [5]. Moreover, immune cells can convert active vitamin D3 [1,25(OH)2D3] from inactive form [25(OH)D3]. Vitamin D with its receptor signal represses autoimmunity.Enhances dendritic cell and regulatory T-cell differentiation to exert anti-inflammatory effect [6].

Individuals having calcifediol levels of ≥40 ng/mL is almost 14times safer than people having vitamin D levels of 20 ng/mL [7]. Vitamin D3 played a prognostic role in covid pandemic. People with serum vitamin D more than 30ng/ml revealed better outcomes [8]. Adding vitamin D in covid treatment regimen proved less ICU care and fatality [9].

Along with vaccinations,if serum 25(OH)D levels can be raised up to 50 ng/mL, further deterioration in new mutation and antibody escaping can be prevented [10].

Cytokine storm and hypercoagulability are the hallmarks of severe COVID-19. Vitamin D3 is accompanied by polyunsaturated fatty acids, polyphenols can exhibit anti-inflammatory as well as antioxidant properties [11].

Covid-19 infection also has deleterious effects on the nervous system. Virus occupies the ACE2 receptor which is widely expressed in the central nervous system also to accelerate dementia and worsen parkinson's disease. SARS-CoV-2 virus invades olfactory and vagus nerve, giving rise to anosmia and related complications which could be lessened by taking 2000–5000 IU of vitaminD per day [12].

Pregnant women exerted severity of covid infection, who did not have additional vitamin D [13].

Vitamin D complementation safeguards against acute respiratory infection, particularly in severely deficient ones [14],where this showed no enhancement in persistent asthma children [15]. Prenatal enhancement with vitamin D also had no role [16].

Low vitamin D levels are associated with major cardiovascular disorders but does not help to mitigate cardiovascular complications nor cancer incidence [17].

Continuous ambulatory peritoneal dialysis (CAPD) patients have low parathyroid levels. Calcitriol provided to these patients may ameliorate the risk of developing stroke [18].

Because of the neuroprotective attitude -vasodilation properties by nitric oxide(NO) and inducing stromal cell derived factor(SDF1α), calcitriol prescription is appreciated in patients with higher risk of developing sub arachnoid hemorrhage and those who are prone to vasospasm [19].

Vitamin D can not help in improvement of diabetes mellitus type 2 not in reserving kidney function in this particular case [20]. Polycystic ovarian syndrome(PCOS) is inversely proportional to calcitriol level. To deduce circulating androgen to alleviate PCOS, calcitriol intake should be advised [21]. Vitamin D acts as cancer biomarkers in some cases. VDR exerts polymorphism to protect against several male-female urogenital cancers and gastrointestinal cancers [22].

Depressive illness is proportional to serum vitamin D concentration because of its surging impact on serotonergic neurotransmitter [23]. Calcitriol in parental form piles up VDR and brain-derived neurotrophic factor (BDNF) in hippocampus of brain, which clearly proves healing of depression and motor malfunction [24].

Vitamin D is a blessing for mankind, playing major life saving roles by incorporating different mechanisms, involving almost all systems of the human body. Further study can enlighten us with unknown facts of this favorable vitamin.

## Please state whether ethical approval was given, by whom and the relevant Judgement's reference number

It is not necessary.

## Please state any sources of funding for your research

None.

## Author contribution

All authors equally contributed to the analysis and writing of the manuscript.

## Please state any conflicts of interest

None.

## Research registration Unique Identifying number (UIN)

1. Name of the registry: Not applicable.
2. Unique Identifying number or registration ID: Not applicable.
3. Hyperlink to your specific registration (must be publicly accessible and will be checked): Not applicable.

## Guarantor

Md Moshiur Rahman.

Assistant Professor, Neurosurgery Department, Holy Family Red Crescent Medical College, 1 Eskaton Garden Rd, Dhaka 1000.

Email: dr.tutul@yahoo.com.

## Annals of medicine and surgery author disclosure form

The following additional information is required for submission. Please note that failure to respond to these questions/statements will mean your submission will be returned. If you have nothing to declare in any of these categories, then this should be stated.
